# Insamgobonhwan Protects Neuronal Cells from Lipid ROS and Improves Deficient Cognitive Function

**DOI:** 10.3390/antiox11020295

**Published:** 2022-01-31

**Authors:** Ji Hye Yang, Cong Duc Nguyen, Gihyun Lee, Chang-Su Na

**Affiliations:** College of Korean Medicine, Dongshin University, Daeho-dong, Naju 58245, Jeollanam-do, Korea; uranus2k@nate.com (J.H.Y.); ducngcong@dsu.ac.kr (C.D.N.)

**Keywords:** Alzheimer, ferroptosis, *Donguibogam*, lipid peroxidation, amyloid beta

## Abstract

Iron is an essential element in the central nervous system that is involved in many of its important biological processes, such as oxygen transportation, myelin production, and neurotransmitter synthesis. Previous studies have observed the selective accumulation of iron in Aβ aggregates and neurofibrillary tangles in the brains of patients with Alzheimer’s disease, and excess of this accumulation is associated with accelerated cognitive decline in Alzheimer’s patients. Emerging evidence suggests that ferroptosis, cell death due to iron accumulation, is a potential therapeutic target for treating Alzheimer’s disease. Insamgobonhwan (GBH) is a well-regarded traditional medicine from *Donguibogam* that possess antioxidant properties and has been suggested to slow the aging process. However, the neuroprotective role of GBH against lipid peroxidation-induced ferroptosis and its positive cognitive effects remain unexplored. Here, we investigated the ability of GBH to protect against RSL3-induced ferroptosis in vitro and to suppress amyloid-β-induced cognitive impairment in vivo. First, we treated HT22 cells with RSL3 to induce ferroptosis, which is an inhibitor of glutathione peroxidase 4 (GPX4) and induces lethal lipid hydroperoxide accumulation, reactive oxygen species (ROS) production, and ferroptotic cell death. GBH treatment inhibited cell death and lipid peroxidation, which were increased by RSL3 administration. In addition, GBH restored the expression of ferroptosis marker proteins, such as GPX4, HO-1 and COX-2, which were altered by RSL3. Next, we examined whether the protective ability of GBH in cells was reproduced in animals. We concluded that GBH treatment inhibited Aβ-induced lipid peroxidation and improved Aβ-induced cognitive impairment in mice.

## 1. Introduction

Alzheimer’s disease, the most common cause of dementia, is characterized by the presence of amyloid-β (Aβ) plaques. Accumulation of iron is observed in Aβ aggregates and neurofibrillary tangles in the brains of patients with Alzheimer’s disease, and excess of this in the brain is associated with accelerated cognitive decline in Alzheimer’s disease patients [1,2]. Iron accumulation in the brain causes ferroptosis, which is a recently discovered form of programmed cell death. Ferroptosis is characterized by mitochondrial reduction and lipid peroxidation, which can be triggered by the inhibition of glutathione peroxidase 4 (GPX4) and by blocking cystine uptake by the system xc-, a cystine/glutamate antiporter [3,4]. Some ferroptosis inhibitors have already shown possible clinical benefits in clinical trials. The metal ion chelating agent deferoxamine and ferroptosis inhibitor ferrostatin-1 may significantly improve the pathology of neurodegenerative diseases in vitro and in vivo [5,6,7,8]. These recent studies provide inspiration to investigate the possibility of targeting ferroptosis in neurodegenerative diseases.

In Asian countries especially Korea, traditional medicine wisdoms have long been a pilar maintaining people health and well-being, both in the past and nowadays; and many researches are on the way to explore and turn these ancient secrets into precious life-saving tools in the future. The distinctive Korean medical philosophies system, was momentously enriched flowing the publication of Donguibogam (translated as “A Precious Mirror of Eastern Medicine”) by the famous Dr. Heo Jun (1546–1615) in 1613. Its’ 25 volumes cover a colossal expanse of medical knowledge. It further systematized the Oriental philosophies in classification and ordering of symptoms and remedies to the targeted disordered organs, rather than the disease itself. This was a radical approach and helped to structuralize the vast diverse traditional medicine viewpoints in East Asia at that time. This intellectual relic has been a source of inspiration for thinkers and artists alike, and is regarded as a classic of Korean vibrant culture and Oriental medicine today. In 2009, in light of its historic and medical values, the United Nations Educational, Scientific, and Cultural Organization (UNESCO) registered the book on its cultural heritage list; and designated the year 2013—400 years after Donguibogam creation—as the ‘Donguibogam Year’ [9,10].

Insamgobonhwan (GBH) is a famous traditional prescription from Donguibogam. This remedy is well regarded as an antioxidant drug candidate and has been suggested to slow down the aging process [11]. The prescription consisted of four herbal species: *Ginseng radix*, *Liriope platyphylla*, *Rehmanniae radix*, and *Asparagus cochinensis*. The ingredient *Rehmanniae radix*, which contains a large number of phenolics, such as rehmannoside A, B and C, versulin, oleanolic acid, and geniposide which all presented excellent antioxidant and anti-inflammatory properties [12]. Especially, 5-hydroxymethylfurfural (5-HMF), a major active component of *Rehmanniae radix preparata*, exhibited in vitro antioxidant activities and was proven to improve cognitive impairment in amyloid beta (Aβ)-induced Alzheimer’s disease [13]. *Ginseng radix* and its’ important ginsenosides: Rg1, Rb1 and Rg3 are famous for their ability to improve brain disorder conditions. They can promote the restoration of neurotransmitters such as serotonin or acetylcholine, hormones such as cortisol, corticosterone, and testosterone, and receptors such as androgen receptors and glucocorticoid receptors. Ginsenoside Rg1 is regarded as the main component of the ginsenosides and has been proven to possess numerous biological functions, including reducing depression [14]. Protodioscin and spicatoside A are respectively famous active compounds of *Asparagus cochinchinensis* and *Liriope platyphylla*, both compounds with proven neuroprotective effects [13,14]. 

Even though GBH possesses valuable chemicals that exhibit antioxidant and neuroprotective functions, there is still a small amount of research regarding the effects of GBH, as a traditional medicine entity, to improve neurodegenerative disorder situations. In particular, the neuroprotective role of GBH against lipid peroxidation-induced ferroptosis and its positive cognitive effects are still unknown. A step from this, the ability of GBH to improve cognitive function deficits in animal models remains limitedly revealed. Therefore, the aim of the present study was to evaluate the neuroprotective effects of GBH in HT22 cells and an immortalized mouse hippocampal neuronal cell line against ferroptosis-mediated cell death. Our results demonstrate that GBH protects HT22 cells through GPX4 recovery and lipid ROS level reduction. Moreover, GBH exhibited signs of cognitive protection in an animal amnesia model caused by Aβ. These results suggest that GBH may be a promising therapeutic agent for the treatment of Alzheimer’s disease.

## 2. Materials and Methods

### 2.1. Materials

Antibodies against phospho-ERK/ERK, phospho-p38/p38, and phospho-JNK/JNK were provided by Cell Signaling Technology (Danvers, MA, USA). HO-1 antibody was purchased from Enzo Life Sciences (Plymouth Meeting, PA, USA). GPX4 antibody was acquired from Proteintech (Chicago, IL, USA) and COX-2 antibody was purchased from Cayman Chemical (Ann Arbor, MI, USA). RSL3 was procured from Selleckchem (Houston, TX, USA). β-actin antibody, tetrazolium dye (MTT), dichloro-dihydro-fluorescein diacetate (DCFH-DA), dimethylsulfoxide (DMSO), Bovine calf serum, and Aβ were obtained from Sigma (St. Louis, MO, USA). Cell staining buffer containing bovine calf serum from BioLegend (San Diego, CA, USA). All herbal medicine ingredients *Liriope platyphylla*, *Asparagus cochinensis*, *Rehmania radix preparata*, *Ginseng radix*, and *Rehmanniae radix* were purchased and used in Korea (Omni-Herb Co., Yerongcheon, Korea). The reference standard chemicals, Rb1 (98.3%), 5-hydroxymethylfurfural (5-HMF) (99.7%), spicatoside A (98.0%), Rg1 (97.0%), Rg3 (97.9%) and Protodioscin (97.8%) were purchased from ChemFace (Wuhan, China).

### 2.2. Preparation of GBH

**Figure 1:**
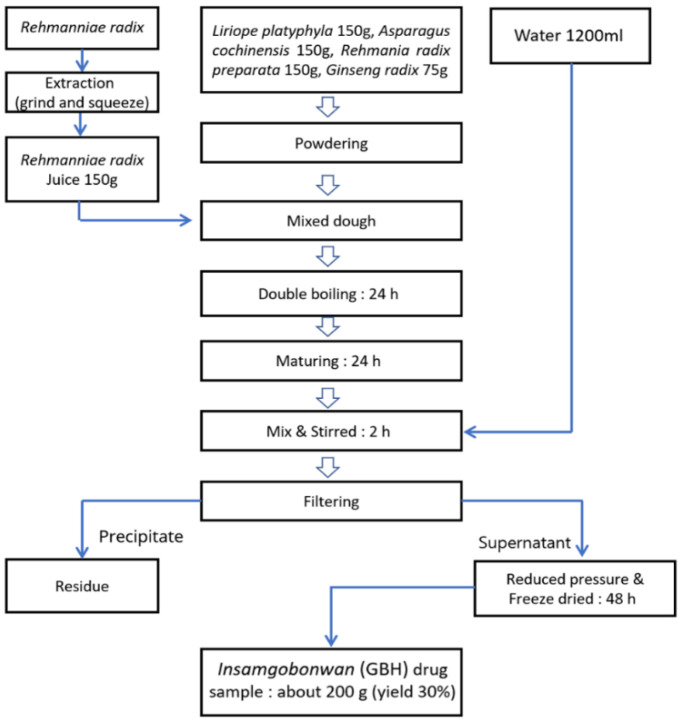
Diagram of the GBH preparation from the raw materials to final freeze-dried product.

The composition and preparation of GBH was done based on the famous Korean traditional medical record *Donguibogam Prescriptions*. The ingredients of the herbal medicine GBH formulation are *Liriope platyphylla* 150 g, *Asparagus cochinchinensis* 150 g, *Rehmania radix preparata* 150 g, *Ginseng radix* 75 g, and *Rehmanniae radix* juice 150 g (obtained by processing *Rehmanniae radix* via a juicer machine (Bando Machinery, Tokushima city, Japan)). *Lyope platyphylla*, *Asparagus cochinensis*, *Rehmania radix preparata*, and *Ginseng radix* were powdered, and mixed with the *Rehmania radix* juice for kneading. The kneaded sample mixture was placed in a pottery vessel for heating. This sample was further processed via the double boiling method in which the pottery was sealed and placed in a boiling water bath (100 °C) for 24 h. Subsequently, this sample was left unattended for 24 h at room temperature, and 600 g of the mixture was obtained from the pottery. This sample was then mixed with 1200 mL of water, stirred for 2 h, and filtered by squeezing it using a cloth bag to obtain the final extract liquid. This extract was concentrated under reduced pressure and then freeze-dried to obtain 200 g (30% yield) of GBH (Figure 1).

### 2.3. HPLC Analysis of the Marker Components in GBH

The eluent solvents used consisted of A: water (10 mM KH_2_PO_4_ solution at pH 4.5), and B: acetonitrile. We used an X Bridge BEH C18 HPLC column (130 Å, 5 µm and 4.6 mm × 250 mm; Waters, Milford, MA, USA). Gradient: 0–15 min: 5% B, 50 min: 70% B, 65 min: 100% B, 65–75 min: 100% B. Flow rate: 0.9 mL/min. Column temperature was maintained at 40 °C. Sample concentration: 50 mg/mL (dissolving solvent 60% ethanol, sonication time—1 h). The injection volume was 10 μL. The standard chemical concentration varied from 1 to 150 μg/mL with similar injection volume of 10 μL. Based on the standard compounds’ concentrations and their respective signal intensity correlations, we established a specific regression equation for each compound to confirm a good linear range and to calculate their concentration in the freeze-dried GBH sample. Analysis was carried out using a Waters HPLC 2965 separation module and a Waters 2996 UV detector.

### 2.4. Culture of HT22 Cells

HT22 cells, an immortalized mouse hippocampal neuronal cell line, were purchased from Merck Millipore (Burlington, MA, USA). DMEM, containing 10% FBS and 50 units/mL penicillin/streptomycin, was used for cell culture, and the cells were grown at 37 °C in a humidified 5% CO_2_ atmosphere.

### 2.5. Cytotoxicity Analysis

To measure the cytotoxicity of GBH, HT22 cells were seeded in wells of experimental plates and treated with GBH for 24 h, and viable cells were stained with MTT as previously reported [15]. Moreover, to evaluate the protective effects of GBH in competitive RSL3-induced cell death, cells were treated with RSL3 and/or GBH for 6 h. Then, we eliminated the media and added 200 μL of DMSO to dissolve the formazan crystals in the wells. Absorbance was measured using a microplate reader (Spectramax, Molecular Devices, Sunnyvale, CA, USA) at 540 nm. Cell viability was defined relative to the vehicle-treated control [viability (% control) = 100 × (absorbance of treated sample)/(absorbance of control)].

### 2.6. ROS Generation Assay

Cellular ROS levels were quantified by measuring the DCF fluorescence intensity [16]. ROS generation was evaluated in HT22 cells treated with RSL3 and/or GBH for 1 h. HT22 cells were stained with DCFH-DA (10 μM) in an incubator maintained at 37 °C for 30 min. The collected HT22 cells were then washed with PBS, and ROS levels were measured using a fluorescence-detecting microplate reader (Molecular Devices) with excitation/emission wavelengths of 485/530 nm. ROS generation was normalized to the protein concentration of each treated sample and was defined relative to the vehicle-treated control.

### 2.7. C11-BODIPY Fluorescence Analysis

After treatment with RSL3 in HT22 cells, the cells were stained with 10 μM C11-BODIPY for 1 h. Cells were harvested by trypsinization and washed with PBS. The intensity of fluorescence in the cells was measured using flow cytometry (Beckman Coulter, Brea, CA, USA). C11-BODIPY fluorescence was determined using the FITC-A channel.

### 2.8. Immunoblot Analysis

The method employed for immunoblotting, including protein extraction, subcellular fractionation, and SDS-PAGE, have been described previously [17]. Samples were separated by gel electrophoresis, and proteins were transferred from the gel to nitrocellulose blotting membranes (GE Healthcare, Chicago, IL, USA) for antibody staining and detection. The blotting membranes were first incubated with the indicated primary antibodies at 4 °C overnight and then incubated with the appropriate HRP-labeled secondary antibodies for 1 h at room temperature. Immunoreactive proteins on the nitrocellulose membranes were visualized using ECL chemiluminescence detection kit (Amersham Biosciences, Buckinghamshire, UK). The β-actin immunoblotting technique was used as a control to verify equal protein loading and the integrity of nuclear fractionation, respectively.

### 2.9. Animals

ICR mice (male; 6 weeks old; 25–30 g) were supplied from Dehan Biolink Co. (Eumseong, Korea) and kept with two mice per cage in pathogen-free conditions (22–26 °C; relative humidity: 50% to 60%) with a 12-h light/dark cycle, and free access to mouse food (Sangyang Co., Osen, Korea) and drink. The mice acclimatized in captivity for five days prior to experiments commencement. Experiments were conducted following the Guide for Care and Use of Laboratory Animals of the National Research Council (NRC, 1996) and were approved by the Committee of Animal Care and Experiment of Dongshin University, Korea (DSU2019-04-02).

### 2.10. In Vivo Drug Administration

Aβ_25–35_ intracerebroventricular (ICV) injections were conducted as described in previous studies [18,19]. Mice received isoflurane anesthesia during the process, and we injected (5 µL) into the right cerebral ventricles using a 28-gauge stainless needle via stereotaxic coordinates (in mm) from the bregma A: −0.22, L: 1.0, V: 2.5, with a flow rate of 5 μL/min. On day 1, the naïve group which received 5 µL of only PBS via ICV, all other groups were injected with 5 µL of prepared PBS solution containing 3 μg of Aβ_25–35_. The drugs were orally administered (PO) daily from days 3 to 14 (3 h before the behavior experiment, days 11–14). Donepezil was used as positive control. PO volume was 0.1 mL. 

The mice population was divided into the following groups (*n* = 8) according to treatments:

(1) PBS ICV + PBS PO
(2) 3 μg Aβ_25–35_ ICV + PBS PO
(3) 3 μg Aβ_25–35_ ICV + donepezil 2 mg/kg PO
(4) 3 μg Aβ_25–35_ ICV + GBH 100 mg/kg PO
(5) 3 μg Aβ_25–35_ ICV + GBH 400 mg/kg PO

### 2.11. Morris Water Maze Experiment

The Morris water maze (MWM) examination was used to assess the efficacy of GBH on spatial learning and memory in mice, as formerly described with slightly adjustments [20]. The MWM apparatus consisted of a circular black-painted water tank (diameter: 120 cm; height: 50 cm) decorated with visual cues (a star, a square, a rectangle, and a circle). The water temperature was kept at 22 ± 2 °C. The tank was virtually divided to four identical quadrants: the southeast, northeast, southwest, and northwest. The platform (diameter: 10 cm; height: 25 cm, black color) was centered in the northwest quadrant. The whole trial process comprised of an adaptive training (day 10, three times a day), hidden platform tests (days 11–14, two trials per day) and a spatial probe test (right after the last hidden platform test on day 14, once, 2 min each). To record the animals swimming behavior, the ANY-maze monitoring software (Stoelting Co., Wood Dale, IL, USA) was used.

### 2.12. Y Maze Behavior Test

Y-maze test was executed on day 14, 5 h right after MWM. This experiment examines the immediate spatial short-term memory. The Y-maze is a three-arm-path (40 cm long, 3 cm wide, and 12 cm high) in which the three paths are equally separated at 120°. The maze floor and walls were black. Mice were first positioned at an arm entrance, and the activities of arm entries were recorded for each mouse over a 5 min period. An actual spontaneous alternation was distinct as entries into three arms was in a successively manner (i.e., ABC, CAB or BCA, but not BAB). To record the animals moving behavior and automatically calculate the results, the ANY-maze monitoring software (Stoelting Co.) was used.

### 2.13. Collection of In Vivo Animal Tissues

After the MWM experiment, on day 11, all mice were anesthetized and their blood and brain samples were collected. Blood serum was collected by centrifugation at 3000 rpm for 10 min at 4 °C. The brains of a subset of animals within each group were used for immunofluorescence analysis (fixed with 4% paraformaldehyde and kept at 4 °C post-fixation), and the hippocampus of the brains were collected and immediately subjected to biochemical or Western blot analysis on the same day.

### 2.14. Lipid Peroxidation Assay In Vivo

The relative malondialdehyde (MDA) levels in cell lysates was assessed using the Lipid Peroxidation (MDA) Assay Kit (ab118970, Abcam, Cambridge, UK). Hippocampal tissue was homogenized in 303 µL lysis solution (buffer + BHT) with a Dounce homogenizer sitting on ice, with 10–15 passes. The mixture was then centrifuged at 13,000× *g* for 10 min and the supernatant was collected for the assay. To prepare blood serum samples, 20 µL of blood serum was mixed with 500 µL of 42 mM H_2_SO_4_ and 125 µL of phosphotungstic acid solution was added, and the precipitate was collected by centrifugation. The total protein levels were normalized among the samples. TBA solution was added to samples and standards, incubated at 95 °C for 60 min, and subsequently cooled in an ice bath for 10 min. Liquid from these tubes was transferred to the wells of a microplate and analyzed with a Versa Max Microplate Reader (Molecular Devices, San Jose, CA, USA).

### 2.15. Doublecortin Immunofluorescence Analysis

Post-fixed brain hemispheres were submerged in 15% sucrose for 12 h and subsequently with 30% sucrose for another 12 h at 4 °C. The brains were frozen using dry CO_2_ ice and sliced into 30 μm sagittal sections. At room temperature, sections were blocked with 6% bovine calf serum and incubated with doublecortin primary antibody (1:200 in cell staining buffer, 2 h), rinsed in cell staining buffer twice for 15 min each, and then incubated with Alexa Fluor 488 secondary antibody (Ex/Em = 490/525 nm, 1:200 in cell staining buffer, 2 h) and two additional rinses in staining buffer for 15 min each. Samples were submerged in Fluoromount™ Aqueous Mounting Medium and shielded with glass coverslips for microscopic imaging. The images were photographed using the Invitrogen EVOS FL Auto Imaging System (Thermo Fisher Scientific, Waltham, MA, USA) with a 20× objective. Cells were counted within an identical area of 100 µm wide across all tissue sections, as shown in the images.

### 2.16. Statistical Analysis

Statistical analysis was carried out as follow: we utilized one-way analysis of variance (ANOVA) to evaluate the significance of the differences between the experimental groups. Subsequently, the Newman-Keuls examination method was applied to assess the significance of differences between the means of different groups. Results are expressed as mean ± SE or ±SD.

## 3. Results

### 3.1. HPLC Analysis of the Marker Compounds in GBH

**Figure 2:**
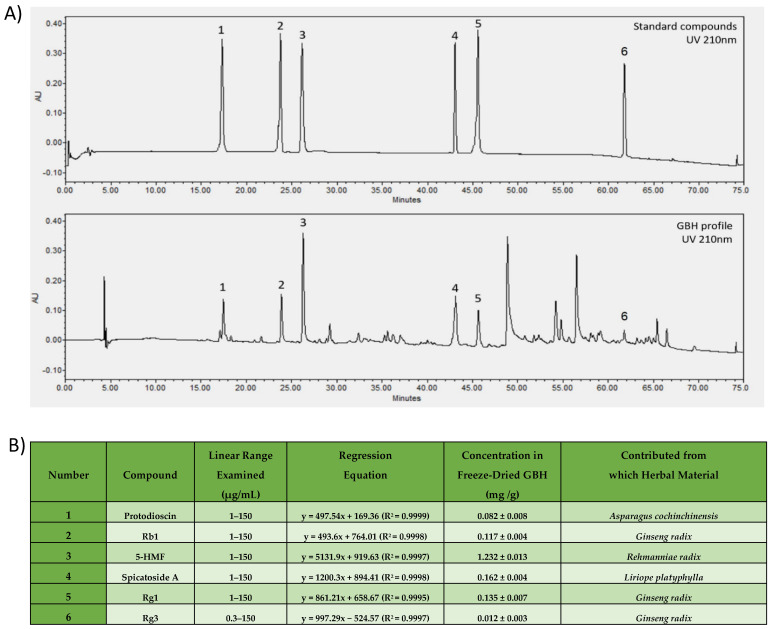
HPLC analysis of GBH. Confirmation of key standard compounds (**A**) Chromatogram of standard compounds and GBH profile. (**B**) Detailed analysis data with references of previous studies that identified the standard compounds in the respective herbal materials, all identified compounds in GBH sample had presented concentrations within the examined linear range 1–150 μg/mL.

We confirmed the presence of Protodioscin in GBH chemical profile (standard compound retention time (S-rt): 17.21 min/GBH compound retention time (G-rt): 17.34 min), which we chose to act as the representative indication for *Asparagus cochinchinensis* in GBH [21,22]. Three ginsenosides Rb1 (S-rt: 23.74 min/G-rt: 23.88 min), Rg1 (S-rt: 45.53 min/G-rt: 45.63 min), and Rg3 (S-rt: 61.80 min/G-rt: 61.82 min) were confirmed to be present in GHB and indicated to be the markers of *Ginseng radix* [23,24]. 5-HMF was detected in the GBH sample (S-rt: 26.09 min/G-rt: 26.22 min) and we chose this compound to be the marker of *Rehmanniae radix* fingerprint in GBH [25,26]. Spicatoside A (S-rt: 43.03 min/G-rt: 43.06 min) was confirmed to be present and was selected to be the marker compound for *Liriope platyphylla* [27,28]. Detailed results of the analysis are presented in Figure 2.

### 3.2. GBH Suppressed Oxidative Stress and Cell Death by RSL3

**Figure 3:**
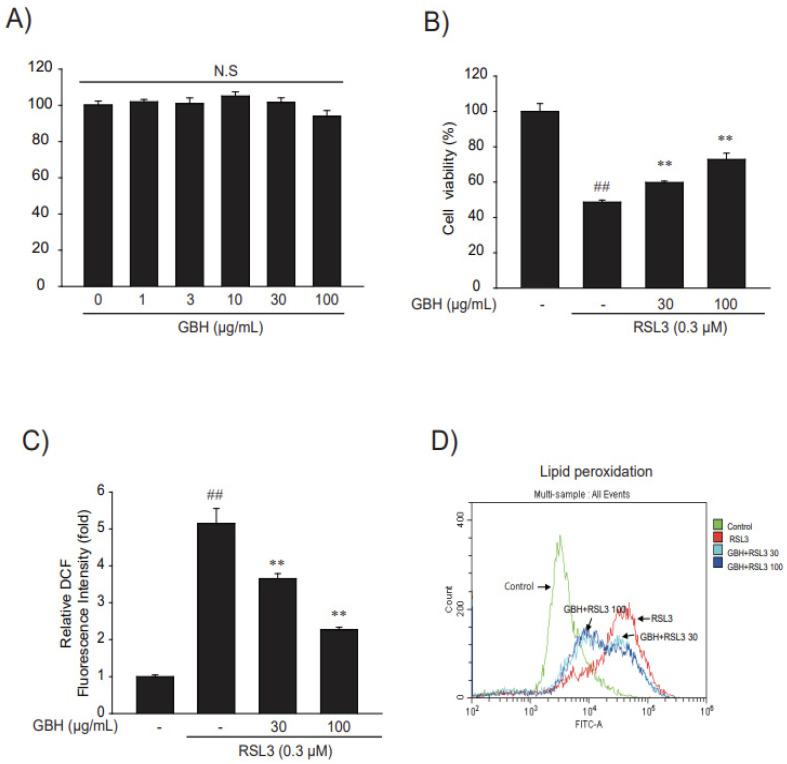
The cytoprotective efficacy of GBH in RSL3-induced HT22 cells. (**A**) The effect of GBH (1–100 μg/mL, 24 h) on the cytotoxicity of HT22 cells was estimated using MTT assay. (**B**) The efficacy of GBH on RSL3 induced cell death. Cells were treated with RSL3 (0.3 μM) and/or 30–100 μg/mL GBH for 6 h. Cell viability was assessed using the MTT assay. (**C**) The efficacy of GBH on RSL3-induced ROS production. The HT22 cells were pretreated with GBH for 1 h and incubated with 0.3 μM RSL3 and/or 30–100 μg/mL GBH for 1 h. Then, cells were stained with 10 μM DCFH-DA at 37 °C for 30 min. Intracellular fluorescence intensities were measured using a fluorescence microplate reader. (**D**) The efficacy of GBH on RSL3-induced lipid peroxidation. The HT22 cells were pretreated with GBH for 1 h and incubated with 0.3 μM RSL3 and/or 30–100 μg/mL GBH for 4 h. Then, cells were stained with 10 μM C11-BODIPY for 1 h. The intensity of fluorescence in the cells was measured using flowcytometry (Beckman-Coulter). Data represent the mean ± S.E. of 3 experiments; ** *p* < 0.01, meaningful versus vehicle-treated control; ^##^ *p* < 0.01, meaningful versus RSL3 alone.

Before we investigated whether GBH has antioxidant and cytoprotective effects, we performed a radical MTT assay to verify the cytotoxicity of GBH in HT22 cells and confirmed that it is safe up to 100 μg/mL (Figure 3A). We then investigated the effect of GBH on the cytotoxic effects of the ferroptosis activator RSL3. RSL3 is an inhibitor of glutathione peroxidase 4 (GPX4) that induces lethal accumulation of lipid hydroperoxides, ROS production, and ferroptotic cell death [29]. GBH treatment inhibited cell death, which was increased by RSL3 treatment (Figure 3B). In addition, treatment with RSL3 of HT22 cells led to ROS generation, whereas pretreatment with GBH significantly prevented ROS formation and lipid peroxidation (Figure 3C,D). These results suggest that GBH has antioxidant properties against ROS and cytoprotective effects in RSL3-induced HT22 cells.

### 3.3. GBH Restored Ferroptosis Marker Protein Expression Changed by RSL3

**Figure 4:**
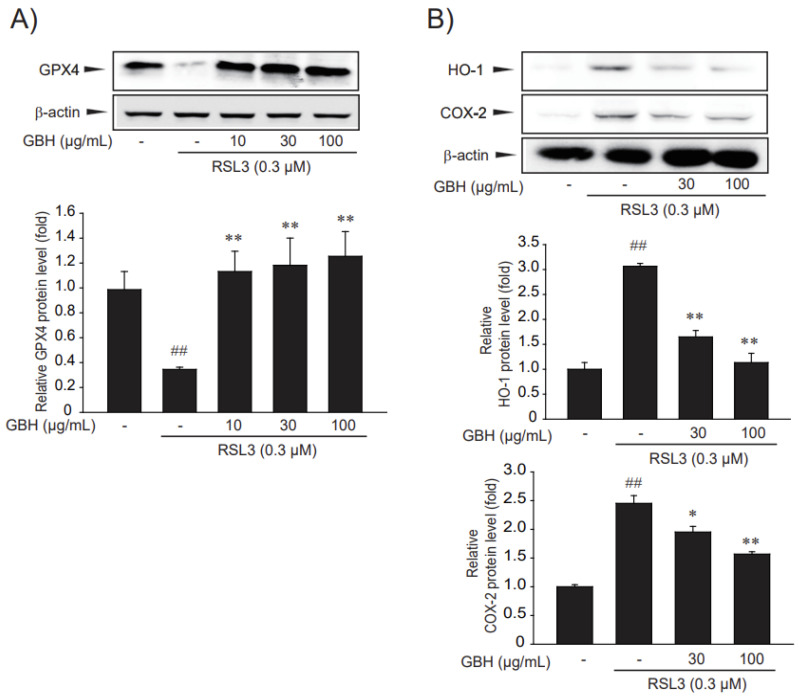
The efficacy of GBH about restored expression of ferroptosis markers in RSL3-induced HT22 cells. (**A**,**B**) The expression of ferroptosis marker proteins, such as GPX4, HO-1 and COX-2, altered due to RSL3. The HT22 cells were pretreated with GBH(GBH) for 1 h and incubated with 0.3 μM RSL3 and/or GBH for 3 h. Protein expressions of GPX4, HO-1 and COX-2 were detected by immunoblot analysis. Data represent the mean ± S.E. of 3 experiments; * *p* < 0.05, ** *p* < 0.01, meaningful versus vehicle-treated control; ^##^ *p* < 0.01, meaningful versus RSL3 alone.

Next, we investigated whether GBH treatment recovered GPX4 expression, which was decreased by RSL3 (Figure 3A). GPX4 is a direct target of RLS3, and its knockdown induces ferroptosis in an iron-, MEK-, and ROS-dependent manner, whereas its overexpression leads to RSL3 resistance [29]. Treatment of HT22 cells with RSL3 led to a decrease in GPX4 expression, whereas pretreatment with GBH significantly restored GPX4 expression (Figure 4A,B). HO-1 and COX-2, like other ferroptosis markers, are upregulated after treatment with ferroptosis activators (RSL3 or erastin) [29,30]. Expectedly, RSL3 increased the expression of other ferroptosis markers, such as HO-1 and COX-2, which were decreased by treatment with GBH (Figure 4). GBH restored the expression of ferroptosis marker proteins in HT22 cells, which were altered by RSL3.

### 3.4. GBH Decreased ERK and JNK Activation Induced by RSL3

**Figure 5:**
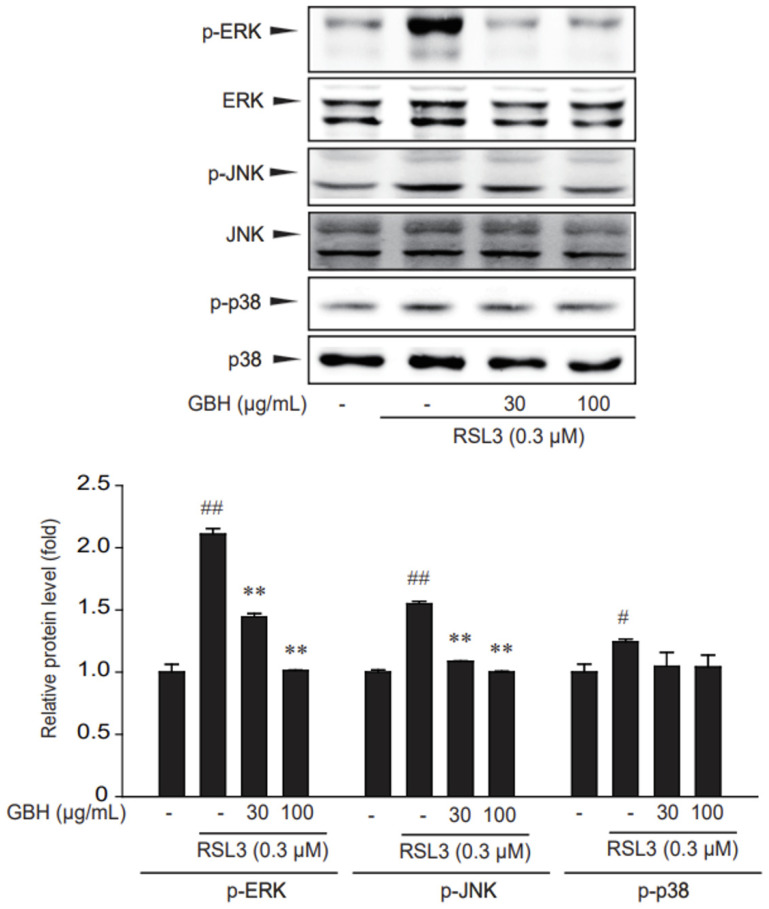
Inhibition of ERK and JNK phosphorylation by GBH in RSL3-induced HT22 cells. Immunoblotting for MAPKs phosphorylation. Cells were treated with 30 or 100 μg/mL GBH 1 h before being incubated with RSL3 for 1 h. The cell lysates were immunoblotted. Data represent the mean ± S.E. of 3 experiments; ** *p* < 0.01, meaningful versus vehicle-treated control; ^#^ *p* < 0.05, ^##^ *p* < 0.01, meaningful versus RSL3 alone.

Lipid peroxidation also induces phosphorylation of MAPKs, including extracellular signal-regulated kinase (ERK), p38 and c-Jun N-terminal kinase (JNK), which are involved in cellular responses to environmental stresses [31,32]. Therefore, we assessed the effect of GBH on the RSL3-induced activation of MAPKs. Phosphorylation of ERK and JNK were specifically suppressed by GBH treatment, whereas p38 phosphorylation was not restrained by GBH treatment (Figure 5). These results indicate that ERK and JNK inhibition by GBH might be involved in its suppressive effect on RSL3-induced cell toxicity.

### 3.5. GBH Inhibits Aβ Induced-Lipid Peroxidation Production In Vivo

**Figure 6:**
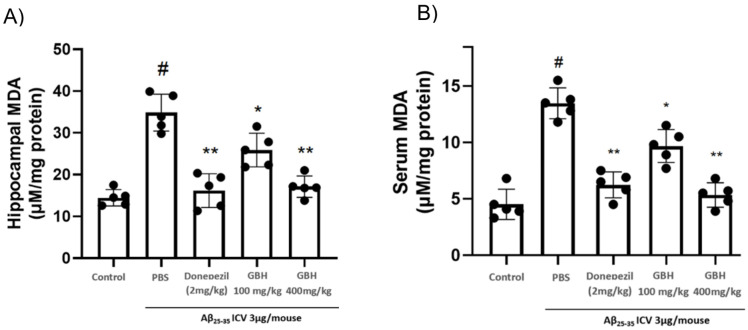
Inhibitory effect of GBH about lipid peroxidation by Aβ in vivo. MDA antioxidant parameters were examined in in vivo hippocampus (**A**) and blood serum (**B**) samples: The results showed that Aβ_25–35_ ICV treatment clearly upregulated oxidative stress levels in the hippocampus, which was inverted by GBH treatment (hippocampus tissue was analyses MDA levels were examined with respective measuring kits). Data are shown as mean ± SD in triplicate. ^#^: *p* < 0.01 vs. control; *: *p* < 0.05 or **: *p* < 0.01 vs. Aβ_1–42_ only-treated group.

The MDA antioxidant parameter is closely related to neurodegenerative diseases and is highly expressed in individuals with neurodegenerative conditions [33]. Our research showed that MDA was increased in hippocampal tissue of Aβ_25–35_ ICV-treated animals by approximately 2.5-fold compared to naïve counterparts, and the treatment with GBH 100 and 400 mg/kg dose-dependently normalized this parameter. At 400 mg/kg GBH significantly reduced oxidative parameter down to equally that of donepezil 2 mg/kg (Figure 6).

### 3.6. GBH Improves Aβ Caused-Cognitive Impairment In Vivo

**Figure 7:**
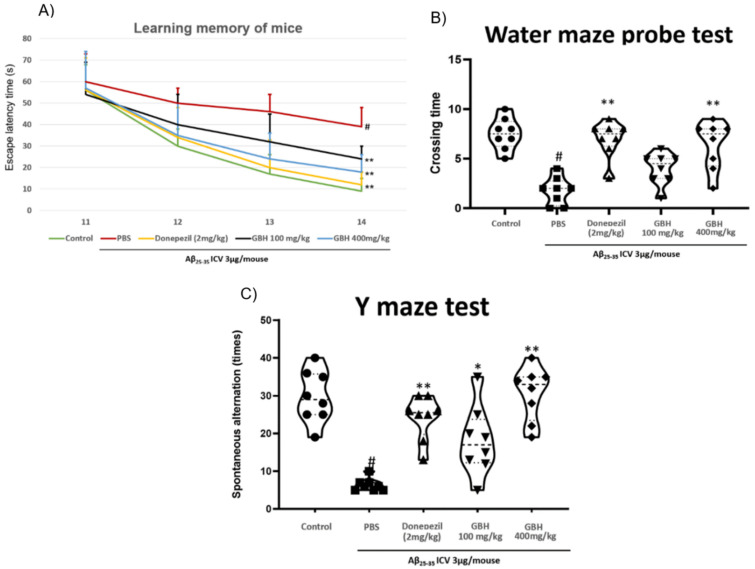
Effect of GBH on the cognitive function of animals suffering from Aβ_25–35_ induced amnesia. (**A**) Morris water maze experiment to examine learning memory function during the training period days 11–14 (each mouse underwent 3 trials a day to find the escape platform in 90 s). (**B**) A probe test was conducted at the end of day 14 to ascertain platform position recall ability in mice (on platform removal, the mice were allowed to swim freely for 120 s to look for platform location and the number of mice crossing the platform position was recorded). (**C**) Y maze experiment carried out on day 14 to assess the short-term memory of animals. Data are shown as mean ± SD in triplicate. ^#^: *p* < 0.01 vs. control; *: *p* < 0.05 or **: *p* < 0.01 vs. Aβ_1–42_ only-treated group.

**Figure 8:**
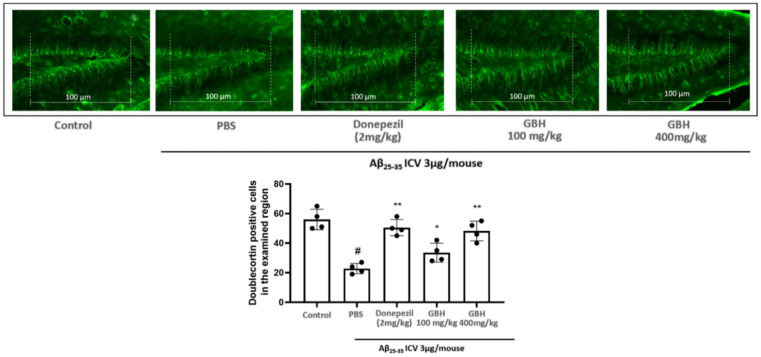
Effect of GBH to recover the impairment caused by Aβ25–35 in animals. DCX Fluorescent immunohistochemical examination was conducted to clarify the effects of GBH (GBH) on Aβ25–35 induced suppression of neurogenesis in the hippocampal dentate gyrus. Cells were counted within an identical area of 100 µm wide across all tissue sections, as shown in the images. Data are shown as mean ± SD in triplicate. #: *p* < 0.01 vs. control; *: *p* < 0.05 or **: *p* < 0.01 vs. Aβ_1–42_ only-treated group.

In the MWM experiment for long-term learning memory ability assessment [34], compared to Aβ_25–35_ injection naïve mice had significantly lengthened the escape latency time of animals up to approximately 4-fold on day 14 (Figure 7A), whereas in the probe test, the crossing time of Aβ_25–35_ induced animals only scored for a third of that of naïve groups. In both these experiments, GBH 100 and 400 mg/kg restored cognitive performance in a dose-dependent manner. A similar pattern was observed in the Y-maze test (Figure 7B), which specifically examined short-term memory [35]. To further confirm the effects of GBH in protecting mice against neurodegenerative stress induced by Aβ_25–35_. We examined brain sections that were stained with DCX. This microtubule-associated protein expressed by migrating immature neurons is a key hallmark to valuating neuronal neurogenesis [36]. The results indicated that treatment with Aβ_25–35_ significantly suppressed the number of DCX-positive neurons in the examined dentate gyrus region, and GBH at both 100 and 400 mg/kg significantly recovered the impairment caused by Aβ_25–35_ (Figure 8). In these experiments, 400 mg/kg GBH treated animals exhibited significantly enhanced behavior indexes as well as doublecortin positive neurons in dentate gyrus area, down to a similar level as the donepezil 2 mg/kg treated group.

## 4. Discussion

GBH is well regarded as an antioxidant medicine and has been suggested to slow the aging process [11]. Regarding the chemical profile of GBH, even though the analyzed chemicals were all reported in each individual herbal ingredient [21,22,23,24,25,26,27,28], the status of each compound in the final GBH freeze-dried drug sample—which had undergone the explained preparation process—was still unknown. The chemical profile we formed up from our analysis, exhibited for the first time some of the most representative major compounds from each ingredient herbal plant, as key chemical analysis markers of GBH. GBH constitutes a mixture of active chemicals from different herbal plants, which were all brought together based on the guidance of the Donguibogam traditional medicine record. This ancient envisioned combine might carry secrets and interested synergetic interactions that are hidden from view. We believe that this chemical study can be the first step for much further downstream studies of GBH’s full constitutive chemicals and their joint biological effects.

During the progression of neuro aging, Aβ is a by-product of the constant degradation of the Aβ precursor protein. Accumulated Aβ naturally forms aggregates over time and has a harmful impact on the central nervous system. This is a key up-stream event in ferroptosis-mediated lipid peroxidation. Aβ neurotoxicity is believed to be a major cause of aging-associated neurodegeneration [37]. Intracerebroventricular injections of Aβ_25–35_ into mouse brains were conducted to induce neural oxidative stress, resulting in cognitive impairments in animals. This model produces a substantial degree of Alzheimer’s advance signs and neurodegeneration symptoms in general [18,38,39]. Aβ_25–35_ aggregates have been shown to develop into ion like canals that insert into cell membranes and promote uncontrollable metal ion influx, which undermines intercellular electrolyte and environment balances [40,41,42]. Similar to full-length Aβ fragments, Aβ_25–35_ can deactivate mitochondrial complex IV, a process that causes mitochondrial respiration abnormalities and produces damaging oxidative stress such as reactive O_2_^•−^ [43,44,45,46,47]. The mitochondrial native superoxide scavengers MnSOD and Cu/ZnSOD react with O_2_^•−^, producing H_2_O_2_. In the presence of free metal ions such as copper (I) or iron (II), H_2_O_2_ is broken down via the Fenton reaction into extremely reactive •OH radicals [47]. It is a canonical role of the mitochondrial failure incident that produces •OH radicals, which contributes to lipid peroxidation and increases MDA levels, a prerequisite for ferroptosis. Additionally, inhibition of the Krebs cycle, electron transport chain, or mitochondrial membrane potential disruption can all provoke ferroptosis under these conditions [48].

We observed that the neuroprotective role of GBH against lipid peroxidation was unknown. This study found that GBH could reduce lipid peroxidation and ERK/JNK activation in RSL3-induced HT22 cells in vitro and undermine cognitive impairment by Aβ_25–35_ in vivo, providing a potential strategy for treating ferroptosis-related neurodegenerative diseases. According to several studies, iron accumulation is observed in the brains of patients with Alzheimer’s disease. Therefore, ferroptosis, cell death due to iron accumulation, is a potential therapeutic target for Alzheimer’s disease [1,2].

Ferroptosis is characterized by the suppression of the phospholipid glutathione peroxidase 4 (GPX4) and subsequent intracellular accumulation of lipid reactive oxygen species (ROS) in an iron-dependent manner [3,29,49,50]. Ferroptosis can be induced by small molecules, including erastin and RSL3. In particular, RSL3 was identified as a potent ferroptosis-triggering agent, which is dependent on the activity of GPX4 [29]. GPX4, as a negative target of RSL3, mediates the suppression of ferroptosis [50]. GBH significantly inhibited RSL3-induced cell viability, total ROS, and lipid ROS (Figure 3) and recovered GPX4 activity (Figure 4A).

We attempted to identify the underlying mechanisms via our present in vitro experiments. The HO-1 protein is a key functional enzyme, that depends on its’ concentration levels, this protein can neutralize oxidative stress, or oppositely promote oxidative mediated via ferroptosis [51]. Due to highly reactive iron and oxidative stress, the role of HO-1 in ferroptosis was recently re-proposed, in which detrimental roles have been demonstrated. Thus, the pro-oxidative activity of HO-1 contributes to ferroptosis induction, which relies on iron accumulation [30,52]. This reflects the fact that, the appropriately activation of nuclear Nrf2/HO-1 signaling pathway helps resist ferroptosis, in contrast, over excessive upregulation of this pathway increases ferroptosis mediated oxidative damage and lipid peroxidation [53,54,55]. Moreover, research has shown that COX-2 is markedly upregulated during ferroptosis, and is considered a biomarker of this process [49,56]. COX-2 is a key enzyme involved in the synthesis of prostaglandins and an essential lipid peroxidation indicator [57]. In addition, studies have shown that COX-2 is markedly upregulated by (1S, 3R)-RSL3 treatment [29]. After RSL3 treatments to HT22 cells, we found that GBH blocked the over expressed levels of HO-1 and reduced this down nearly as that level of normal non-treatments cells group. Levels of COX-2 also had been significantly reduced by GBH. Thus, reducing lipid peroxidation sensitivity (Figure 4B).

The MAPK signaling pathway can respond to environmental stresses, such as temperature, pH, and redox status [58]. Lipid peroxidation also induces phosphorylation of MAPKs, including ERK, p38 and JNK, which are involved in cellular responses to environmental stresses [31,32,58]. In our study, pretreatment with GBH significantly reduced ERK/JNK phosphorylation, which might reduce the occurrence of ferroptosis (Figure 5).

As exhibited in our experiments, the MDA levels in both blood and hippocampal tissues of animals were drastically elevated after Aβ_25–35_ ICV injection, and GBH treatment effectively reduced lipid peroxidation levels, restored neurogenesis, and reinforced animal cognitive function. These results suggest that GBH may be a promising therapeutic agent for the treatment of Alzheimer’s disease.

## Data Availability

The data presented in this study are available in article.

## References

[1] Good P.F., Perl D.P., Bierer L.M., Schmeidler J. (1992). Selective Accumulation of Aluminum and Iron in the Neurofibrdary Tangles of Alzheimer’s Disease: A Laser Microprobe (LAMMA) Study. Ann. Neurol. Off. J. Am. Neurol. Assoc. Child Neurol. Soc..

[2] Ayton S., Wang Y., Diouf I., Schneider J.A., Brockman J., Morris M.C., Bush A.I. (2020). Brain iron is associated with accelerated cognitive decline in people with Alzheimer pathology. Mol. Psychiatry.

[3] Dixon S.J., Lemberg K.M., Lamprecht M.R., Skouta R., Zaitsev E.M., Gleason C.E., Patel D.N., Bauer A.J., Cantley A.M., Yang W.S. (2012). Ferroptosis: An Iron-Dependent Form of Nonapoptotic Cell Death. Cell.

[4] Xie Y., Hou W., Song X., Yu Y., Huang J., Sun X., Kang R., Tang D. (2016). Ferroptosis: Process and function. Cell Death Differ..

[5] Fine J.M., Kosyakovsky J., Baillargeon A.M., Tokarev J.V., Cooner J.M., Svitak A.L., Faltesek K.A., Frey W.H., Hanson L.R. (2020). Intranasal deferoxamine can improve memory in healthy C57 mice, suggesting a partially non-disease-specific pathway of functional neurologic improvement. Brain Behav..

[6] Mahoney-Sánchez L., Bouchaoui H., Ayton S., Devos D., Duce J.A., Devedjian J.-C. (2021). Ferroptosis and its potential role in the physiopathology of Parkinson’s Disease. Prog. Neurobiol..

[7] Tian Y., Lu J., Hao X., Li H., Zhang G., Liu X., Li X., Zhao C., Kuang W., Chen D. (2020). FTH1 Inhibits Ferroptosis Through Ferritinophagy in the 6-OHDA Model of Parkinson’s Disease. Neurotherapeutics.

[8] Zhang Y., He M.-L. (2017). Deferoxamine enhances alternative activation of microglia and inhibits amyloid beta deposits in APP/PS1 mice. Brain Res..

[9] Kim S., Song B.-K., Won J.-H. (2016). Historical Medical Value of Donguibogam. J. Pharmacopunct..

[10] Seo S.-Y., Sharma V.K., Sharma N. (2003). Mushroom tyrosinase: Recent prospects. J. Agric. Food Chem..

[11] Kurek-Górecka A., Komosinska-Vassev K., Rzepecka-Stojko A., Olczyk P. (2020). Bee Venom in Wound Healing. Molecules.

[12] Liu C., Ma R., Wang L., Zhu R., Liu H., Guo Y., Zhao B., Zhao S., Tang J., Li Y. (2017). Rehmanniae Radix in osteoporosis: A review of traditional Chinese medicinal uses, phytochemistry, pharmacokinetics and pharmacology. J. Ethnopharmacol..

[13] Liu A., Zhao X., Li H., Liu Z., Liu B., Mao X., Guo L., Bi K., Jia Y. (2014). 5-Hydroxymethylfurfural, an antioxidant agent from Alpinia oxyphylla Miq. improves cognitive impairment in Aβ1–42 mouse model of Alzheimer’s disease. Int. Immunopharmacol..

[14] Hou W., Wang Y., Zheng P., Cui R. (2020). Effects of Ginseng on Neurological Disorders. Front. Cell. Neurosci..

[15] Jin S.H., Yang J.H., Shin B.Y., Seo K., Shin S.M., Cho I.J., Ki S.H. (2013). Resveratrol inhibits LXRα-dependent hepatic lipogenesis through novel antioxidant Sestrin2 gene induction. Toxicol. Appl. Pharmacol..

[16] Han J.Y., Cho S.S., Yang J.H., Kim K.M., Jang C.H., Park D.E., Bang J.S., Jung Y.S., Ki S.H. (2015). The chalcone compound isosalipurposide (ISPP) exerts a cytoprotective effect against oxidative injury via Nrf2 activation. Toxicol. Appl. Pharmacol..

[17] Gu S.M., Park M.H., Hwang C.J., Song H.S., Lee U.S., Han S.B., Oh K.W., Ham Y.W., Song M.J., Son D.J. (2015). Bee venom ameliorates lipopolysaccharide-induced memory loss by preventing NF-kappaB pathway. J. Neuroinflamm..

[18] Fang F., Liu G.-T., Liu F.F.-T. (2006). Protective effects of compound FLZ on beta-amyloid peptide-(25-35)-induced mouse hippocampal injury and learning and memory impairment1. Acta Pharmacol. Sin..

[19] Amin F.U., Shah S.A., Kim M.O. (2017). Vanillic acid attenuates Aβ1-42-induced oxidative stress and cognitive impairment in mice. Sci. Rep..

[20] Leggio G.M., Miranda M.-I., Provensi G., Wang C.-H., Li S.P., Wang Y.W., Qi S.L., Zhang Y.P., Ding W.Z., Lin Q.Y. (2018). Analogous β-Carboline Alkaloids Harmaline and Harmine Ameliorate Scopolamine-Induced Cognition Dysfunction by Attenuating Acetylcholinesterase Activity, Oxidative Stress, and Inflammation in Mice. Front. Pharmacol..

[21] Kim M., Kim W.-B., Koo K.Y., Kim B.R., Kim D., Lee S., Son H.J., Hwang D.Y., Kim D.S., Lee C.Y. (2017). Optimal Fermentation Conditions of Hyaluronidase Inhibition Activity on Asparagus cochinchinensis Merrill by Weissella cibaria. J. Microbiol. Biotechnol..

[22] Jaiswal Y., Liang Z., Ho A., Chen H., Zhao Z. (2014). A Comparative Tissue-specific Metabolite Analysis and Determination of Protodioscin Content in Asparagus Species used in Traditional Chinese Medicine and Ayurveda by use of Laser Microdissection, UHPLC-QTOF/MS and LC-MS/MS. Phytochem. Anal..

[23] Sun B.S., Gu L.J., Fang Z.M., Wang C.Y., Wang Z., Sung C.K. (2009). Determination of 11 Ginsenosides in Black Ginseng Developed from Panax ginseng by High Performance Liquid Chromatography. Food Sci. Biotechnol..

[24] Mohanan P., Subramaniyam S., Mathiyalagan R., Yang D.-C. (2018). Molecular signaling of ginsenosides Rb1, Rg1, and Rg3 and their mode of actions. J. Ginseng Res..

[25] Lin A.-S., Qian K., Usami Y., Lin L., Itokawa H., Hsu C., Morris-Natschke S.L., Lee K.-H. (2008). 5-Hydroxymethyl-2-furfural, a clinical trials agent for sickle cell anemia, and its mono/di-glucosides from classically processed steamed Rehmanniae Radix. J. Nat. Med..

[26] Won T.H., Liao L., Kang S.S., Shin J. (2014). Simultaneous analysis of furfural metabolites from Rehmanniae radix preparata by HPLC-DAD–ESI-MS. Food Chem..

[27] Kim J.E., Go J., Lee H.S., Hong J.T., Hwang D.Y. (2019). Spicatoside A in red Liriope platyphylla displays a laxative effect in a constipation rat model via regulating mAChRs and ER stress signaling. Int. J. Mol. Med..

[28] Park G., Parveen A., Kim J.-E., Cho K.H., Kim S.Y., Park B.J., Song Y.-J. (2019). Spicatoside A derived from Liriope platyphylla root ethanol extract inhibits hepatitis E virus genotype 3 replication in vitro. Sci. Rep..

[29] Yang W.S., SriRamaratnam R., Welsch M.E., Shimada K., Skouta R., Viswanathan V.S., Cheah J.H., Clemons P.A., Shamji A.F., Clish C.B. (2014). Regulation of Ferroptotic Cancer Cell Death by GPX4. Cell.

[30] Kwon M.-Y., Park E., Lee S.-J., Chung S.W. (2015). Heme oxygenase-1 accelerates erastin-induced ferroptotic cell death. Oncotarget.

[31] Poursaitidis I., Wang X., Crighton T., Labuschagne C., Mason D., Cramer S.L., Triplett K., Roy R., Pardo O., Seckl M.J. (2017). Oncogene-Selective Sensitivity to Synchronous Cell Death following Modulation of the Amino Acid Nutrient Cystine. Cell Rep..

[32] Angelova P.R., Esteras N., Abramov A.Y. (2021). Mitochondria and lipid peroxidation in the mechanism of neurodegeneration: Finding ways for prevention. Med. Res. Rev..

[33] Peña-Bautista C., Vento M., Baquero M., Cháfer-Pericás C. (2019). Lipid peroxidation in neurodegeneration. Clin. Chim. Acta.

[34] Vorhees C.V., Williams M. (2006). Morris water maze: Procedures for assessing spatial and related forms of learning and memory. Nat. Protoc..

[35] Kraeuter A.K., Guest P.C., Sarnyai Z. (2019). The Y-Maze for Assessment of Spatial Working and Reference Memory in Mice. Methods in Molecular Biology.

[36] Couillard-Despres S., Winner B., Schaubeck S., Aigner R., Vroemen M., Weidner N., Bogdahn U., Winkler J., Kuhn H.-G., Aigner L. (2005). Doublecortin expression levels in adult brain reflect neurogenesis. Eur. J. Neurosci..

[37] Cheignon C., Tomas M., Bonnefont-Rousselot D., Faller P., Hureau C., Collin F. (2018). Oxidative stress and the amyloid beta peptide in Alzheimer’s disease. Redox Biol..

[38] Stepanichev M.Y., Zdobnova I.M., Zarubenko I.I., Moiseeva Y.V., Lazareva N.A., Onufriev M.V., Gulyaeva N.V. (2004). Amyloid-β(25–35)-induced memory impairments correlate with cell loss in rat hippocampus. Physiol. Behav..

[39] Mantha A.K., Moorthy K., Cowsik S.M., Baquer N.Z. (2006). Neuroprotective Role of Neurokinin B (NKB) on β-amyloid (25–35) Induced Toxicity in Aging Rat Brain Synaptosomes: Involvement in Oxidative Stress and Excitotoxicity. Biogerontology.

[40] Lee J., Kim Y.H., Arce F.T., Gillman A.L., Jang H., Kagan B.L., Nussinov R., Yang J., Lal R. (2017). Amyloid β Ion Channels in a Membrane Comprising Brain Total Lipid Extracts. ACS Chem. Neurosci..

[41] Zaretsky D.V., Zaretskaia M.V. (2021). Flow cytometry method to quantify the formation of beta-amyloid membrane ion channels. Biochim. Biophys. Acta (BBA)-Biomembr..

[42] Ekinci F.J., Linsley M.-D., Shea T.B. (2000). β-Amyloid-induced calcium influx induces apoptosis in culture by oxidative stress rather than tau phosphorylation. Mol. Brain Res..

[43] Canevari L., Clark J.B., Bates T. (1999). β-Amyloid fragment 25-35 selectively decreases complex IV activity in isolated mitochondria. FEBS Lett..

[44] Lahmy V., Long R., Morin D., Villard V., Maurice T. (2015). Mitochondrial protection by the mixed muscarinic/σ1 ligand ANAVEX2-73, a tetrahydrofuran derivative, in Aβ25–35 peptide-injected mice, a nontransgenic Alzheimer’s disease model. Front. Cell. Neurosci..

[45] Clementi M.E., Marini S., Coletta M., Orsini F., Giardina B., Misiti F. (2005). Aβ(31–35) and Aβ(25–35) fragments of amyloid beta-protein induce cellular death through apoptotic signals: Role of the redox state of methionine-35. FEBS Lett..

[46] Luque-Contreras D., Carvajal K., Toral-Rios D., Franco-Bocanegra D., Campos-Peña V. (2014). Oxidative Stress and Metabolic Syndrome: Cause or Consequence of Alzheimer’s Disease?. Oxid. Med. Cell. Longev..

[47] Swomley A.M., Förster S., Keeney J.T., Triplett J., Zhang Z., Sultana R., Butterfield D.A. (2014). Abeta, oxidative stress in Alzheimer disease: Evidence based on proteomics studies. Biochim. Biophys. Acta (BBA)-Mol. Basis Dis..

[48] Gao M., Yi J., Zhu J., Minikes A., Monian P., Thompson C.B., Jiang X. (2019). Role of Mitochondria in Ferroptosis. Mol. Cell.

[49] Seibt T.M., Proneth B., Conrad M. (2019). Role of GPX4 in ferroptosis and its pharmacological implication. Free Radic. Biol. Med..

[50] Angeli J.P.F., Schneider M., Proneth B., Tyurina Y.Y., Tyurin V.A., Hammond V.J., Herbach N., Aichler M., Walch A., Eggenhofer E. (2014). Inactivation of the ferroptosis regulator Gpx4 triggers acute renal failure in mice. Nat. Cell Biol..

[51] Chiang S.-K., Chen S.-E., Chang L.-C. (2019). A Dual Role of Heme Oxygenase-1 in Cancer Cells. Int. J. Mol. Sci..

[52] Chang L.-C., Chiang S.-K., Chen S.-E., Yu Y.-L., Chou R.-H., Chang W.-C. (2018). Heme oxygenase-1 mediates BAY 11–7085 induced ferroptosis. Cancer Lett..

[53] Wei R., Zhao Y., Wang J., Yang X., Li S., Wang Y., Yang X., Fei J., Hao X., Zhao Y. (2021). Tagitinin C induces ferroptosis through PERK-Nrf2-HO-1 signaling pathway in colorectal cancer cells. Int. J. Biol. Sci..

[54] Peng W., Zhu Z., Yang Y., Hou J., Lu J., Chen C., Liu F., Pi R. (2021). N2L, a novel lipoic acid-niacin dimer, attenuates ferroptosis and decreases lipid peroxidation in HT22 cells. Brain Res. Bull..

[55] Wu W.L., Papagiannakopoulos T. (2020). The Center Cannot Hold: NRF2 Battles Ferroptosis in the 3rd Dimension. Mol. Cell.

[56] Lei P., Bai T., Sun Y. (2019). Mechanisms of Ferroptosis and Relations with Regulated Cell Death: A Review. Front. Physiol..

[57] Kumagai T., Matsukawa N., Kaneko Y., Kusumi Y., Mitsumata M., Uchida K. (2004). A lipid peroxidation-derived inflammatory mediator: Identification of 4-hydroxy-2-nonenal as a potential inducer of cyclooxygenase-2 in macrophages. J. Biol. Chem..

[58] Hattori K., Ishikawa H., Sakauchi C., Takayanagi S., Naguro I., Ichijo H. (2017). Cold stress-induced ferroptosis involves the ASK 1-p38 pathway. EMBO Rep..

